# Cluster analysis allowed to identify antifungal drugs that retain efficacy against *Candida albicans* isolated from patients with inflammatory diseases of the soft tissues of the maxillofacial area

**DOI:** 10.3389/froh.2024.1446045

**Published:** 2024-09-06

**Authors:** Mariia Faustova, Volodymyr Dobrovolskyi, Galina Loban’, Yevhenii Bereza, Aleksandra Kotelnikova, Oleksandr Dobrovolskyi

**Affiliations:** ^1^ Department of Microbiology, Virology and Immunology, Poltava State Medical University, Poltava, Ukraine; ^2^ Department of Surgery with Course of Dentistry, Pirogov Memorial National Medical University, Vinnytsia, Ukraine; ^3^ Department of Prosthetics Dentistry With Implantology, Poltava State Medical University, Poltava, Ukraine

**Keywords:** *Candida albicans*, resistance, susceptibility, antifungal drugs, cluster analysis

## Abstract

Physicians are increasingly prescribing antifungal drugs empirically to treat hospital-acquired infections quickly. This makes it obvious that fungal infections require more attention and systematic monitoring of resistance among them. The aim of the study was to identify antifungal drugs that retain their efficacy against *C. albicans* isolates. There were 17 clinical isolates of *Candida albicans* obtained from patients and tested for susceptibility to antifungal drugs using the standard double dilution method. Amphotericin B, fluconazole, itraconazole, micafungin, and posaconazole were used in the study. To determine the groups of antimycotics to which the studied microorganisms retain sensitivity, a hierarchical cluster analysis was performed using the Ward's method. The tested representatives of the genus *Candida* showed the lowest sensitivity to fluconazole. The efficacy of amphotericin B and itraconazole was almost at the same level. In turn, micafungin and posaconazole showed the best results against *C. albicans isolates*. Ward's cluster analysis combined the results of *C. albicans* susceptibility to fluconazole, micafungin and itraconazole by the highest mathematical similarity. Amphotericin B and posaconazole were combined into one cluster due to their better efficacy against *Candida albicans* isolates.

## 1 Introduction

*Candida* spp. are dimorphic fungi that colonize the oral cavity, genitals and gastrointestinal tract of healthy individuals. However, on the other hand, representatives of this genus of microorganisms are among the top five causative agents of hospital-acquired infections worldwide (1). Along with vaginal and oral candidiasis, *Candida* spp. can cause invasive infections of deep tissues and bloodstream in immunocompromised individuals (2–4). Currently, there is evidence of the role of these fungi in the development of severe postoperative complications in patients in surgical and intensive care units (5, 6). Among more than 200 *Candida* species, only 15 play an important role in the development of human infections, the most common—*Candida albicans, Candida glabrata, Candida parapsilosis, Candida tropicalis*, and *Candida krusei* (1). It is worth noting that, according to the literature, *C. albicans* is the most common among patients in Europe (more than 50.0% of cases) and North America (40.0%) (7). The WHO emphasizes the significant danger to public health from *C. albicans* isolates. After all, the global mortality rate is up to 50.0%, and about 5.0% of infections show repeated growth after long-term treatment with antifungals (8).

Reports of the acquisition of fluconazole resistance in representatives of the genus *Candida* began to appear in the 90s of the last century and had become more frequent recently (9). Laboratory monitoring in the USA indicates the development of resistance to fluconazole, as the most widely used drug, among isolates of *C. albicans* at the level of 2.0%. However, some non-albicans species achieve resistance to fluconazole in 93.0% of populations, for example *C. auris* (10). Along with this, recent studies by Korean scientists showed the development of fluconazole resistance in 33.0% of *Candida* spp (11). A similar situation exists in European countries. Thus, during 2019–2022 in Spain, resistance to fluconazole among *Candida* spp. was recorded in the range of 8.0–13.0% (12). Given these negative trends, two representatives of the genus *Candida* (*C. albicans* and *C. auris*) were included by the WHO in 2022 in the list of fungi of the critical priority group (8).

The rapid acquisition of resistance to antifungal drugs by *Candida* spp. is evident, given the frequency of fungal infections and the slow pace of the development of new antifungals (6, 13). The situation has become especially complicated in the world, including in Ukraine, against the background of the COVID−19 pandemic and an active armed conflict (14–16). After all, physicians increasingly prescribe antibiotics as well as antifungal drugs empirically for the rapid treatment of nosocomial infections (17). This makes it obvious that fungal infections require more attention and systematic monitoring of their resistance. However, the mechanisms of antimicrobial resistance of *Candida* spp*.* are less well understood compared to bacteria or viruses (2, 18).

The aim of the work was to determine antifungal drugs that retain their effectiveness against *C. albicans* isolates.

## 2 Methods

### 2.1 Ethics

Written informed consent was obtained from each subject after a detailed explanation of the aim and protocol of the study, which was conducted in accordance with the ethical principles set forth in the Declaration of Helsinki for Ethical Principles for Medical Research Involving Human Subjects. The study was approved by the commission on biomedical ethics of the Poltava State Medical University (minutes #210 dated November 23, 2022).

### 2.2 Inclusion and exclusion criteria

**Figure 1:**
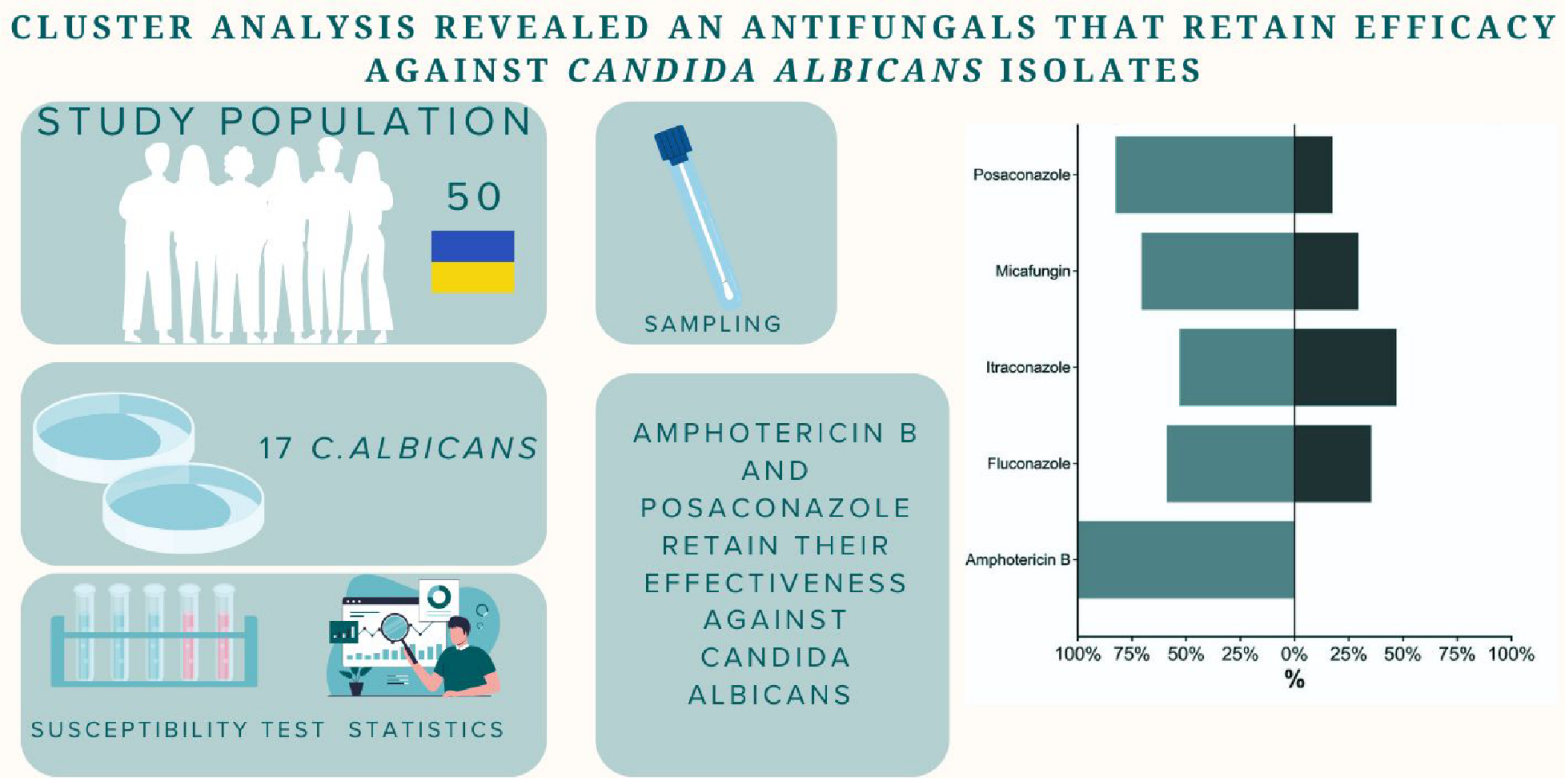
Graphical abstract.

The study included 50 patients who were treated for infectious and inflammatory diseases of the soft tissues of the maxillofacial area in the Department of Oral and Maxillofacial Surgery of the Poltava Regional Center for Dentistry—Dental Clinical Polyclinic of the Poltava Regional Council (Ukraine) during 2022–2023 (Figure 1).

The criteria for the inclusion of patients in the study was the confirmed diagnosis of L00-L08—Infectious diseases of the skin and subcutaneous tissue according to ICD-10, subject to consent to participate in the study. Exclusion criteria were non-compliance with the diagnosis L00-L08 according to ICD-10, pregnancy, diabetes, presence of congenital or acquired immunodeficiency, mental disorders, taking antibiotics the day before collecting specimens, and refusal to participate in the study.

### 2.3 Collecting biological material

Samples were taken from the site of the infected surgical wound of the maxillofacial area with sterile probe swabs placed in AMIES transport medium. Microorganisms were inoculated on Sabouraud Gentamicin Chloramphenicol 2 agar (BioMerioux, France) at 35°C for 48 h. The final identification of the isolates was carried out by morphological, tinctorial and biochemical properties with automatic bacteriological analyzer Vitek 2 compact (BioMerioux, France) according to the manufacturer's instructions.

### 2.4 Antifungal agents

The study used amphotericin B 0.5% (Ampholip, Bharat Sirams&Vaccines Limited, India), fluconazole 5.0% (Fluconazole-Darnytsia (PJSC “Pharmaceutical Firm” Darnytsia”, Ukraine), itraconazole 10.0% (Itrakon, JSC “Farmak”, Ukraine), micafungin 5.0% (MicafunginAccord, AccordHealthcareLimited, Velika Britain), posaconazole 4.0% (Posaconazole-Teva, Genepharm SA.JSC “Grindex”, Israel). Antifungals were obtained from reliable commercial sources.

### 2.5 Susceptibility testing

The standard double dilution method was used to determine the sensitivity of the *C. albicans* isolates to antifungal drugs. The minimum inhibitory concentrations (MIC) of antifungal drugs against the studied microorganisms were determined.

Two-fold serial dilutions of the test preparations were prepared in RPMI 1640 with 2% glucose in accordance with the recommendations of the EUCAST standard (v. 10.0 valid from 2020 to 02-04). The suspension of microorganisms was prepared by suspending the overnight culture of *C. albicans* in the nutrient medium with a final concentration of 5 × 10^5^ CFU/ml, which is equivalent to a turbidity of 0.5 according to the McFarland standard. The microplates with the prepared dilutions were incubated at 35°C for 20 h, followed by determination of the optical density of the well contents in comparison with the control without antifungal drugs using a spectrophotometer (wavelength 600 nm). The MIC is the highest dilution of the antifungal drug under study that prevented visible growth of the studied isolates (19).

### 2.6 Statistical analysis

For descriptive statistics, we used mean, standard deviation, median, minimum, maximum frequency, and percentage.

To determine the groups of antimycotics to which the studied microorganisms retain sensitivity, a hierarchical cluster analysis was performed using the Ward's method. The method consists in combining closely spaced clusters and creating small clusters. The distance between clusters was the increment of the sum of squared distances of objects to the centers of the clusters obtained as a result of their association. Analysis of variance methods were used to estimate the distances between clusters. At each step of the algorithm, the following two clusters were merged, which led to the minimum increase in the objective function, i.e., the intra-group sum of squares (20).

Statistical analysis was performed using standard software IBM SPSS Statistics version 22.0. and GraphPad Prism Software 10.1.0.

## 3 Results

The study revealed that the tested *C. albicans* isolates showed the lowest susceptibility to fluconazole, as its MIC was the highest (Supplementary Table 1). In turn, the MICs of amphotericin B and itraconazole were almost at the same level, being 11.4 and 18.8 times lower than the result of fluconazole, respectively. It is worth noting that the minimum concentrations of micafungin and posaconazole in relation to the tested microorganisms were the lowest. The MICs of micafungin and posaconazole were 197.9 and 94.0 times significantly lower, respectively, compared to the MIC of fluconazole (*p* < 0.05).

**Figure 2:**
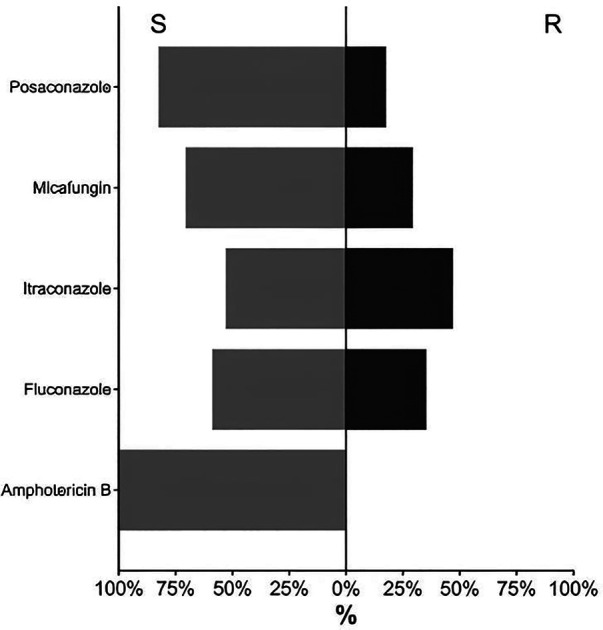
Pattern of *C. albicans* susceptibility to antifungal agents (*n* = 17).

Evaluating the obtained results, according to the clinical breakpoints of EUCAST for *C. albicans*, it was found that all tested isolates (Abs. 17; 100.0%) were susceptible to amphotericin B (Figure 2). 82.4% (Abs. 14) of the yeast-like fungi isolated from patients showed sensitivity to posaconazole, and only three isolates (17.6%) were classified as resistant to this antifungal agent. 70.6% (*n* = 12) of *C. albicans* isolates retained susceptibility to micafungin, and 52.9% (*n* = 9) to itraconazole. The susceptibility to fluconazole of the studied microorganisms was 58.8% (Supplementary Table 1). That is, the percentage of resistant *C. albicans* isolates to the main antifungal drugs ranged from 17.6% to 47.1%, with the exception of amphotericin B.

**Figure 3:**
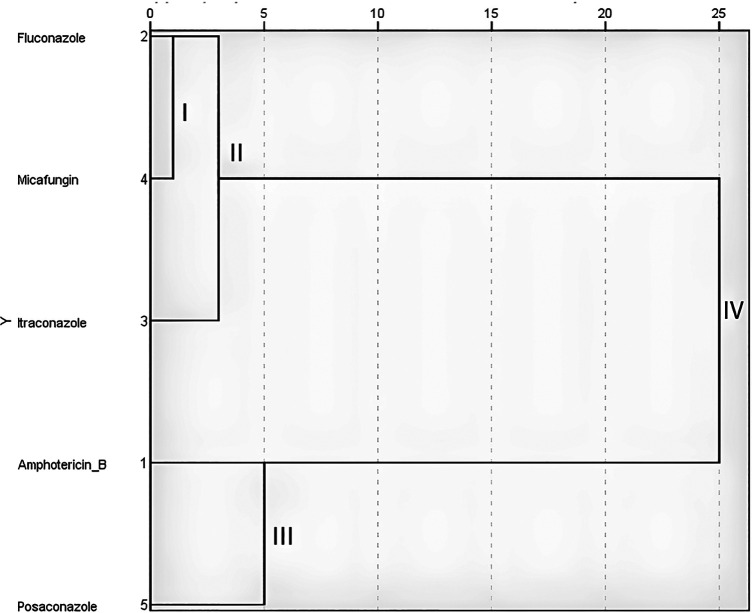
Screen image of the IBM SPSS Statistics software, dendrogram of the cluster analysis of *C. albicans* (*n* = 17) sensitivity to antifungals according to Ward's method.

The cluster analysis of the sensitivity of the studied *С. albicans* to antifungal drugs by the Ward method revealed the formation of cluster I, which united fluconazole and micafungin (Figure 3). At the second stage of clustering, itraconazole joined them to form cluster II. This indicates the greatest mathematical similarity of the results of *C. albicans* susceptibility to fluconazole, micafungin and itraconazole, since clusters I and II were formed at Euclidean distances of 1 and 3, respectively. A little later (Euclidean distance 5), cluster III was formed, which combined the results of *Candida* susceptibility to amphotericin B and posaconazole, confirming their mathematical neighborhood. Further stages of clustering did not lead to the unification of the results until the Euclidean distance of 25 was reached, when all the results were combined into the last single cluster IV. This confirmed the lack of statistical similarity between clusters II and III.

## 4 Discussion

Undoubtedly, the development of severe mycoses of maxillo-facial area caused by *C. albicans* is directly related to a lot of conditions including the immunodeficiency states of patients (21). However, there is currently evidence of frequent genetic and physiological changes in yeast-like fungal cells with the transition of *C. albicans* from a conditionally pathogenic to a pathogenic species (22). A number of *in vitro* studies indicate changes in the protein composition and genetic regulation of *C. albicans* metabolism, which provide them with new virulence factors, make them more aggressive, invasive and resistant to antifungal drugs (22, 23). An example of this is the emergence of resistance among *Сandida* spp. to the most common antifungal drug, fluconazole. Mutations leading to changes in the drug target and the pathways of sterol and ergosterol biosynthesis increase the resistance of *C. albicans* to fluconazole (24). Moreover, sexual recombination between different fungal cells contributes to the transfer of resistance mechanisms, which in turn leads to the formation of highly fluconazole-resistant populations and cross-resistance to other azoles (24, 25). This may explain our results, since itraconazole and fluconazole showed the lowest activity against the studied *C. albicans* isolates. In addition, their grouping into a single cluster at the second stage of clustering indicates a close relationship and similarity of their action. Earlier studies on 1,400 isolates of *С. albicans* showed similarity in the results of their susceptibility to fluconazole and itraconazole. Moreover, as in our study, itraconazole demonstrated slightly better efficacy (26). The average MIC values of fluconazole obtained during this study (3.76 ± 3.320 mg/L) exceed those in some countries. Thus, in Brazil and Thailand, the MIC of fluconazole for candida was 1.0 mg/L. In addition, the MIC of fluconazole against *C. albicans* was recorded at 4 mg/L in Iran (27, 28).

Unexpectedly, in our study, more than 70.0% of clinical isolates of *С. albicans* showed phenotypic signs of sensitivity to micafungin, while literature data in different countries indicated a much lower rate. For example, Danish researchers identified resistance in more than 50.0% (29). Despite the fact that micafungin has a different mechanism of antifungal action and is recommended as a first-line antifungal drug in Europe, we observed a statistical similarity of its efficacy results against *C. albicans* with first-generation azoles (30). Recently, American scientists proved the development of cross-resistance of *Candida* spp. to micafungin and fluconazole within a week against the background of echinocandin monotherapy (31). Despite the fact that the MIC of micafungin for the studied microorganisms was one of the lowest, according to clinical EUCAST data, the rate of resistance development to this drug was one of the highest. Taking into account the above, it becomes obvious that itraconazole, fluconazole and micafungin are united in one cluster of the least effective antifungal agents against *C. albicans* according to the results of statistical analysis.

Amphotericin B, an antifungal drug from the polyene class, showed the best result, which corresponds to the results of studies by German scientists (32). This drug binds to ergosterols of the cell membrane of the fungal cell, embedding into it. This promotes the formation of ion channels through which intracellular components are released and the cell dies (33). However, when prescribing it as part of therapy, it is necessary to take into account the main side effect—nephrotoxicity. For this purpose, it is worth paying attention to liposomal variants of amphotericin (34). It is worth noting that the second-generation triazole Posaconazole demonstrated efficacy against *C. albicans*, statistically similar to Amphotericin B. This new drug of the triazole class in clinical trials demonstrates an advantage over other representatives of this class of antifungal agents (35).

## 5 Conclusions

The Ward's cluster analysis showed the highest mathematical similarity of the results of *Candida albicans* susceptibility to fluconazole, micafungin and itraconazole as antifungal drugs with the lowest effect. Amphotericin B and Posaconazole retain their efficacy against *Candida albicans* isolates and are promising for prescription as part of complex therapy of patients.

## Data Availability

The original contributions presented in the study are included in the article/Supplementary Material, further inquiries can be directed to the corresponding author.
