# Supplementary material for: Cluster analysis allowed to identify antifungal drugs that retain efficacy against Candida albicans isolated from patients with inflammatory diseases of the soft tissues of the maxillofacial area
Source: Front Oral Health. 2024 Sep 6;5:1446045. doi: 10.3389/froh.2024.1446045 (PMC11412946; doi:10.3389/froh.2024.1446045)
Supplement: Supplementary file 1 [file Table1.docx]

Supplementary Table 1

Quantitative and qualitative indicators of *C. albicans* sensitivity to antimycotics, mg/L

| No | Amphotericin B | | Fluconazole | | Itraconazole | | Micafungin | | Posaconazole | |
| --- | --- | --- | --- | --- | --- | --- | --- | --- | --- | --- |
|  | MIC | S/I/R | MIC | S/I/R | MIC | S/I/R | MIC | S/I/R | MIC | S/I/R |
| *C. albicans* 01 | 0.125 | S | 2.0 | S | 0.016 | S | 0.008 | S | 0.016 | S |
| *C. albicans* 02 | 0.25 | S | 8.0 | R | 0.5 | R | 0.03 | R | 0.125 | R |
| *C. albicans* 03 | 0.125 | S | 1.0 | S | 0.016 | S | 0.016 | S | 0.016 | S |
| *C. albicans* 04 | 0.125 | S | 1.0 | S | 0.016 | S | 0.016 | S | 0.016 | S |
| *C. albicans* 05 | 0.25 | S | 8.0 | R | 0.5 | R | 0.03 | R | 0.06 | S |
| *C. albicans 06* | 0.125 | S | 1.0 | S | 0.03 | S | 0.016 | S | 0.016 | S |
| *C. albicans* 07 | 1.0 | S | 8.0 | R | 0.5 | R | 0.03 | R | 0.016 | S |
| *C. albicans* 08 | 0.125 | S | 2.0 | S | 0.125 | R | 0.016 | S | 0.016 | S |
| *C. albicans* 09 | 0.125 | S | 1.0 | S | 0.016 | S | 0.008 | S | 0.016 | S |
| *C. albicans* 10 | 1.0 | S | 8.0 | R | 0.5 | R | 0.03 | R | 0.06 | S |
| *C. albicans* 11 | 0.125 | S | 1.0 | S | 0.016 | S | 0.016 | S | 0.125 | R |
| *C. albicans* 12 | 0.125 | S | 0.5 | S | 0.016 | S | 0.008 | S | 0.016 | S |
| *C. albicans* 13 | 1.0 | S | 8.0 | R | 0.5 | R | 0.03 | R | 0.03 | S |
| *C. albicans* 14 | 0.25 | S | 2.0 | S | 0.03 | S | 0.016 | S | 0.06 | S |
| *C. albicans* 15 | 0.5 | S | 4.0 | I | 0.5 | R | 0.016 | S | 0.016 | S |
| *C. albicans* 16 | 0.125 | S | 0.5 | S | 0.016 | S | 0.016 | S | 0.016 | S |
| *C. albicans* 17 | 0.25 | S | 8.0 | R | 0.125 | R | 0.016 | S | 0.125 | R |
| Average | 0.33±0.330 | | 3.76±3.320 | | 0.20±0.230 | | 0.019±0.008* | | 0.04±0.042* | |

Notes: MIC - minimum inhibitory concentrations; S – susceptible, I – susceptible in increase exposure, R – resistant; * - validity of the result difference with respect to the fluconazole result.
